# A multidisciplinary expert consensus on optimal acute adult psychiatric inpatient care in Hong Kong

**DOI:** 10.3389/fpubh.2025.1719409

**Published:** 2025-12-15

**Authors:** Dennis Chak Fai Ma, Kit Ping Chan, Janelle Yorke, Grace Wing Ka Ho, Wai Ching Yan, Chi Wing Law, Wai Song Yeung, Tarun Bastiampillai, Adrian P. Mundt, Sherry Kit Wa Chan

**Affiliations:** 1 School of Nursing, The Hong Kong Polytechnic University, Hung Hom, Hong Kong SAR, China; 2 Department of Psychiatry, School of Clinical Medicine, LKS Faculty of Medicine, The University of Hong Kong, Pokfulam, Hong Kong SAR, China; 3 Kwai Chung Hospital, Kwai Chung, Hong Kong SAR, China; 4 Department of Psychiatry, Kowloon Hospital, Kowloon City, Hong Kong SAR, China; 5 Department of Psychiatry, Queen Mary Hospital, Pokfulam, Hong Kong SAR, China; 6 Department of Psychiatry, Pamela Youde Nethersole Eastern Hospital, Chai Wan, Hong Kong SAR, China; 7 College of Medicine and Public Health, Flinders University, Flinders Medical Centre, Adelaide, SA, Australia; 8 Centro de Investigación Biomédica, Facultad de Medicina, Universidad Diego Portales, Santiago, Chile; 9 Department of Psychiatry and Mental Health, Facultad de Medicina Norte, Hospital Clínico Universidad de Chile, Santiago, Chile

**Keywords:** general adult psychiatry, optimal psychiatric inpatient care, psychiatric beds, expert consensus, Delphi study

## Abstract

**Introduction:**

The number of psychiatric inpatient beds has been significantly decreased since the deinstitutionalisation movement began in the 1950s. Subsequent efforts have largely focused on developing psychiatric services based on community mental health models. However, optimal clinical care standards require minimum inpatient care capacities and effective integration between inpatient and community psychiatric care.

**Methods:**

To establish the optimal number of psychiatric inpatient beds and factors related to optimal adult psychiatric inpatient care in Hong Kong, an online, two-round Delphi survey was used to collect expert opinions from experienced psychiatric nurses, a social worker, and psychiatrists working in public psychiatric inpatient or community settings. Medians and interquartile ranges were calculated for numeric responses. Thematic analysis was used to qualitatively categorise the themes of contextual factors for optimal bed numbers and optimal inpatient care. Expert consensus was reached after two rounds of the online survey according to a priori consensus definitions.

**Results:**

In total, 47 experts were invited to participate. Twenty-nine participated in the panel of the first round and 22 in the second round of the Delphi study. 68% had > 20 years post-registration experience. Expert consensus revealed an optimal median number of 68 psychiatric inpatient beds per 100,000 population (interquartile range = 11) and a nurse–patient ratio of 1:4. Two themes were identified relating to recommendations for psychiatric inpatient bed numbers and three themes relating to optimal acute adult psychiatric inpatient services in Hong Kong, respectively. One theme relating to optimal bed numbers was the presence of service needs in the population, such as the prevalence of severe mental illnesses and substance use disorders.

**Conclusion:**

Experts in Hong Kong reached consensus on the need for more psychiatric beds than currently available to achieve a balanced care system. This study also presents consensus on a nurse-inpatient ratio that would require investments in more staff and has the potential to improve the quality of inpatient treatment and efficiency of care.

## Introduction

Since the 1950s, deinstitutionalisation movements in psychiatry began in the UK, the US, and other Western high-income countries, fuelled by the combination of the availability of more effective psychiatric medications and more recovery-oriented care models. In Hong Kong, deinstitutionalisation started later in the 2000s with gradually reduced psychiatric bed numbers. Apart from the continuous development of community psychiatric care to better suit the needs of the patients, financial benefits became one of the major drivers of bed number reduction (1–7). For example, the Royal College of Psychiatrists (8) found that the relative cost of one adult acute psychiatric bed was equivalent to 44 caseloads in a community mental health team in the UK. However, with over half a century of such development, a revisit of optimal psychiatric inpatient care requirements alongside modern community psychiatric care is needed. Further, little is known about how the deinstitutionalisation movement originated in Western countries suits culturally different settings.

The first aspect of optimal psychiatric inpatient care is the quantity of facilities and the numbers of acute care beds. Although the optimal number of psychiatric beds likely varies across different regions based on society’s needs, studies have attempted to obtain consensus among different experts. For example, an influential report in 2008 by the Treatment Advocacy Center in the US compiled the opinions of 15 experts in psychiatry and suggested that the minimum number of public psychiatric beds was 50 per 100,000 population, with ranges identified between 40 and 60 (9). In a recent worldwide Delphi study, including 12 Western Pacific countries (5), the optimal median number of beds identified was 60 per 100,000 population. According to the latest Organisation for Economic Co-operation and Development (OECD) data (10), the median and IQR of the actual numbers of psychiatric beds in 34 mostly high-income countries were 64 and 51 per 100,000 population. However, a recent report showed the number of psychiatric beds per 100,000 population in Hong Kong was 49 per 100,000 population (11). This suggests that Hong Kong, which adopted the deinstitutionalisation model in the 2000s by gradually reducing the psychiatric bed numbers (i.e., a reduction of 40%, from 81 per 100,000 in 2000), may be below the international standards for optimal numbers of psychiatric beds.

The second aspect is the quality of care, including staff resources. The functions of acute psychiatric care include assessment, diagnosis, risk management, crisis intervention, and treatment of acute phases of mental illnesses (12). The clinical guidelines published by the National Institute for Health and Care Excellence (NICE) (13, 14) outline eight quality standards and measures for adult inpatient psychiatric services, such as daily one-to-one contact with staff for at least an hour and daily meaningful and culturally appropriate activities. The Saudi Patient Safety Center (SPSC) published a White paper in collaboration with the International Council of Nurses (ICN) on nurse staffing levels in 2019, advocating that the recommended nurse-to-patient ratio in psychiatric inpatient units should be 1:6 (15). Although California in the US passed a law (AB-394) regarding a minimal nurse–patient ratio in 1999 with 1:6 in psychiatric units (16, 17), the other states in the US and the UK did not endorse any single operational nurse staffing models (18, 19). Quality of care in adult inpatient settings requires additional functional and structural resources. Considerable research has also been conducted to evaluate inpatient intervention programmes to ensure the quality of acute psychiatric care, such as ‘Safe Ward’, ‘Six Core Strategies’, and body-worn cameras, focusing specifically on risk management and preventing patient violence (20–24). Shortening the length of stay (LoS) was regarded as one of the service indicators reflecting the psychiatric inpatient care capacity and efficiency (25). Research revealed that apart from sociodemographic and clinical characteristics of inpatients, the number of beds and hospital size were positively associated with LoS (26–28). However, an appropriate length of stay with effective non-pharmacological and recovery-based interventional approaches was stressed for sustained recovery upon discharge (29). Johnson et al. (30) also highlighted the need for improving the healing environment, using more non-pharmacological approaches, and operationalising the safe staffing level in acute psychiatric inpatient care.

In Hong Kong, although the total cost of services for psychiatric inpatient services contributed to 55.9% of the mental health services provided by the Hospital Authority in 2022 (31), the growth in financial recurrent investment in psychiatric inpatient care is comparatively less than that of community psychiatric care in recent fifteen years. For example, the total expenditure on psychiatric inpatient services (cost per patient day × number of patient days) from fiscal years 2008–2009 (one year before the community psychiatric services development period) to 2023–2024 only increased 1.2 times, after controlling for inflation, whereas the total expenditure on extended hospital psychiatric services (excluding day hospital and psychogeriatric outreach) (cost per psychiatric outreach visit × number of psychiatric outreach) increased 3.8 times in the same period (32). Moreover, the government expenditure to support the Integrated Community Centre for Mental Wellness (ICCMW), a territory-wide community mental health service operated by non-government organisations, also increased 2.3 times, from fiscal years 2009–2010 to 2023–2024, after controlling for inflation (33), as well as other large-scale charity funds such as the Hong Kong Jockey Club Charities Trust contributing to community mental health programmes. The major adult psychiatric service developments in the recent decade were the introduction of Common Mental Disorders Clinics (CMDCs), the ICCMW programme (34), peer support services, and the strengthening of the Early Assessment Service for Young People with Early Psychosis (EASY) (35, 36) and case management programmes (37) with minimal focus on the quality improvement and development of general adult inpatient services. In fact, only a few additional recurrent resources for service enhancement programmes were allocated to general adult psychiatric inpatient services, despite that about 60% of all service users of public psychiatric services were adults in the fiscal year of 2020–2021 (31).

Although there was no apparent waitlist volume for psychiatric emergency admission and no long waiting time for new urgent case booking at psychiatric specialist outpatient clinics in Hong Kong (11, 38), multiple aversive incidents, such as manslaughter of a co-patient during hospitalisation (39) and random killing of two young women by a discharged individual with paranoid schizophrenia (40) occurred recently and revealed the need for comprehensively reviewing the optimal adult psychiatric inpatient care model to provide quality and evidence-based care of general adult psychiatric inpatients, alongside with the existing community psychiatric care facilities. Inpatient psychiatric care remains one of the most important psychiatric care components for some populations during the acute stage of the illness. Arranging for inpatient care is just the first step and the quality of care in the hospital setting is crucial for recovery. The current study aims to establish a local consensus on the optimal number of inpatient psychiatric bed numbers and the care model for adults in need of psychiatric hospitalisation in Hong Kong.

## Methods

### Study design

A Delphi survey approach was used in this study. We conducted two rounds of online surveys to collect and synthesise views on the experiences and expectations of optimal acute inpatient psychiatric care from experienced psychiatrists, psychiatric nurses, and social workers. Ethical approval was obtained from the respective institutional review boards of the participating institutions (UW 22–138 & KW/FR-22-068(175–07)).

### Participant and setting

A systematic review of 80 healthcare studies using the Delphi method (41) revealed that the median and interquartile range (IQR) of the numbers of expert panel members were 17 and 20, respectively. In addition, an umbrella review of the Delphi technique by Niederberger and Spranger (42) found that a low double-digit sample size was acceptable for forming a panel to obtain expert consensus on clinical ideas. Based on the systematic reviews, 30 expert panel members were aimed to be recruited for this Delphi survey.

The inclusion criteria were (i) working in academic institutions, the Hospital Authority public hospitals, or NGOs on a full-time basis or retired ≤ 2 years ago, and (ii) ≥ 10 years post-registration experience. The exclusion criterion was no working experience in adult psychiatry. In addition, professionals with senior roles and advanced clinical expertise were preferentially approached, given their experience in clinical and management positions. Eligible experts were identified by the first and last authors, and a senior psychiatric nurse of a local psychiatric hospital to represent professionals of different roles in adult inpatient psychiatric services. A list of potential participants was prepared after verbal expressions of interest. An email invitation with an online Qualtrics questionnaire link was sent to each potential participant, and the questionnaire could only be commenced after supplying online informed consent.

### Online Delphi questionnaire

The Delphi questionnaire was adapted and modified from a previous international Delphi survey on the optimal number of psychiatric beds (5). The definition of psychiatric beds followed the World Health Organisation (43). It included all beds in mental health hospitals, in psychiatric units, in general hospitals, and in forensic inpatient units, whereas, beds in community residential facilities, beds exclusively serving recovery and rehabilitation treatments or individuals with alcohol and substance abuse disorders or intellectual disability without accompanying mental disorder diagnoses in the community were excluded. After discussions within the research team and with international collaborators, three sections of questions were added to determine optimal acute psychiatric inpatient care. The questionnaire consisted of nine open-ended questions, four numeric responses, and a sociodemographic information questionnaire. Apart from asking about the optimal number of psychiatric beds, we sought opinions on contextual factors and recommendations for acute adult inpatient psychiatric services in Hong Kong, the optimal hospital length of stay (LoS) for different diagnoses according to an International Statistical Classification of Diseases and Related Health Problems, Tenth Revision (ICD-10) (44), and the optimal daytime nurse-to-patient ratio in acute adult psychiatric wards. The expert panel members were advised to offer rationales for each of their responses in the online Delphi questionnaire.

### Procedures

In each round, the expert panel members were expected to complete the questionnaire within 1 month. In the second round, after the removal of duplicates, the anonymous responses of contextual factors with supporting rationales in the first round from each member were shared with the other members for their review. They also rated these responses on a 5-point Likert scale (‘strongly agree’, ‘agree’, ‘neither agree or disagree’, ‘disagree’, and ‘strongly disagree’; or ‘essential’, ‘important’, ‘do not know/depends’, ‘unimportant’, and ‘should not be considered’) to show their level of agreement with these items on contextual factors. The median and IQR of the numeric items were also prepared for the members to review and then reach consensus. A reminder email was sent to each member 1 week before the deadline for each round of the survey. The first round of the survey was conducted from June 2022 to February 2023, and the second round was conducted from March 2023 to April 2023.

### Data analysis

The medians, minima, first and third quartiles, IQRs, maxima, means, and SDs were computed for the numeric responses. The frequencies and percentages of the responses were summarised for the contextual factor open-ended questions. Expert consensuses were reached after two rounds of the survey, according to *a priori* consensus definitions of ≥ 85% agreement with the two favourable responses (i.e., ‘strongly agree’ and ‘agree’) on items regarding contextual factors and ≥ 85% of numeric responses falling within the first and third quartiles of the responses in the first round. These consensus thresholds were based on the previous international Delphi survey (5), which was considered stringent and practical in reaching consensus on this topic. Tables were used to present these findings across the first and second rounds, and two boxplots to illustrate the expert consensus on the number of psychiatric beds and optimal LoS by diagnosis in Hong Kong were produced using R software (45). Thematic analysis was carried out by the first author for coding and theme categorisation of the contextual factors (qualitative data) of consensus items in the second round (46, 47). The consensus on contextual factors and their corresponding themes was presented in tables and confirmed by all authors. Sensitivity analyses of the contextual factors were performed to control for the low base effect by lowering the consensus criterion from ≥ 85% to ≥ 75% to construct themes on recommendations for inpatient psychiatric bed numbers and optimal acute adult psychiatric inpatient services in Hong Kong.

## Results

In the first round, 29 out of 47 experts completed the online questionnaire, and the response rate was 62%. In the second round, 22 members finished the questionnaire, and the response rate was 76%. Table 1 describes the sociodemographic information of the expert panel members involved in the two rounds and shows that the panel comprised both young and experienced experts with different professional backgrounds and a range of roles, areas of expertise, and work experiences in various institutions. The second round of expert panel members predominately worked in an inpatient setting and most held a master’s degree.

**Table 1:** Sociodemographic information of expert panel members.

		First round	Second round
		(*n* = 29)	(*n* = 22)
Age group			
	34 or younger	4 (14%)	3 (14%)
	35–44	9 (31%)	5 (23%)
	45–54	9 (31%)	7 (32%)
	55–64	7 (24%)	7 (32%)
Gender			
	Male	14 (48%)	14 (64%)
	Female	15 (52%)	8 (36%)
Highest educational level			
	Bachelor’s degree	5 (17%)	1 (5%)
	Master’s degree	22 (76%)	18 (82%)
	Doctorate/PhD degree	2 (7%)	3 (14%)
Professionals			
	Psychiatrist	4 (14%)	3 (14%)
	Psychiatric nurse	24 (83%)	18 (82%)
	Social worker	1 (3%)	1 (5%)
Area of expertise			
	Clinical	17 (59%)	11 (50%)
	Administration/policy/other	7 (24%)	6 (27%)
	Academia/research	5 (17%)	5 (23%)
Roles			
	Primarily frontline duty	19 (66%)	12 (55%)
	Primarily management duty	10 (35%)	10 (46%)
Place of work			
	Public psychiatric hospital	17 (59%)	11 (50%)
	Psychiatric unit in a general hospital	4 (14%)	5 (23%)
	Community psychiatric centre	1 (3%)	1 (5%)
	Academic institution	7 (24%)	5 (23%)
Working nature			
	Inpatient	21 (72%)	16 (73%)
	Community	8 (28%)	6 (27%)
Post-registration working experience (years)			
	10–14	10 (35%)	6 (27%)
	15–19	1 (3%)	1 (5%)
	20–24	8 (28%)	5 (23%)
	25–29	1 (3%)	3 (14%)
	30+	9 (31%)	7 (32%)
Experience in psychiatry (years)			
	0–9	1 (3%)	0
	10–14	11 (38%)	7 (32%)
	15–19	4 (14%)	2 (9%)
	20–24	4 (14%)	4 (18%)
	25–29	3 (10%)	2 (9%)
	30+	6 (22%)	7 (32%)
Experience in adult psychiatry (years)			
	0–9	4 (14%)	5 (23%)
	10–14	11 (38%)	5 (23%)
	15–19	3 (10%)	4 (18%)
	20–24	3 (10%)	2 (9%)
	25–29	3 (10%)	1 (5%)
	30+	5 (17%)	5 (23%)

### Expert consensus on the number of psychiatric beds in Hong Kong

**Figure 1:**
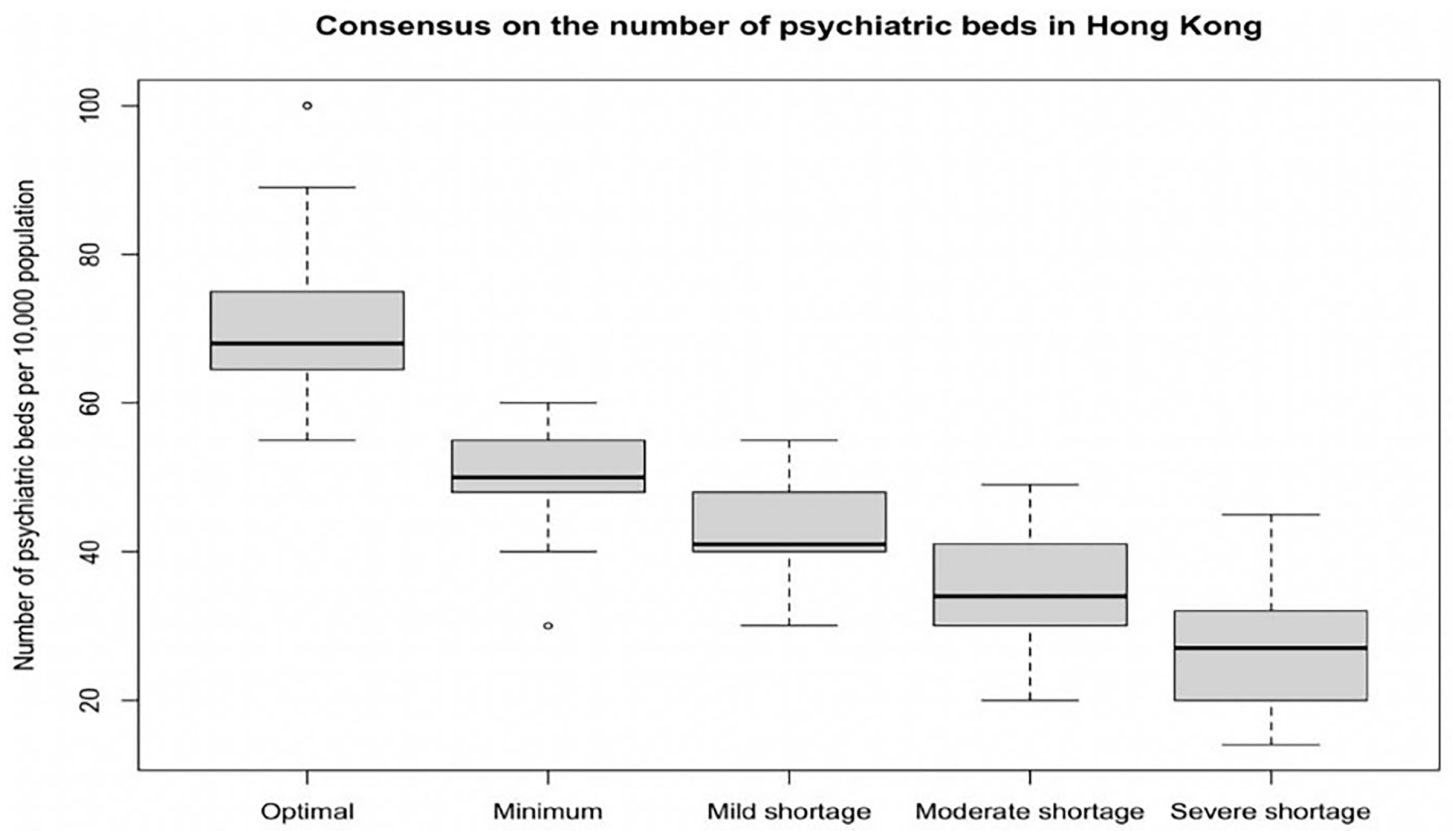
Expert consensus on the number of psychiatric beds in Hong Kong.

Supplementary Table 1 lists the expert consensus on the optimal, minimum, and shortage numbers of adult psychiatric beds in Hong Kong. The medians and IQRs of the optimal number of beds, the minimum number of beds, and the mild, moderate, and severe shortage in the number of beds were 68 (11), 50 (7), 41 (7), 34 (10), and 27 (11) per 100,000 population, respectively. Figure 1 visualises these findings in a boxplot.

### Expert consensus on the LoS by diagnosis in Hong Kong

**Figure 2:**
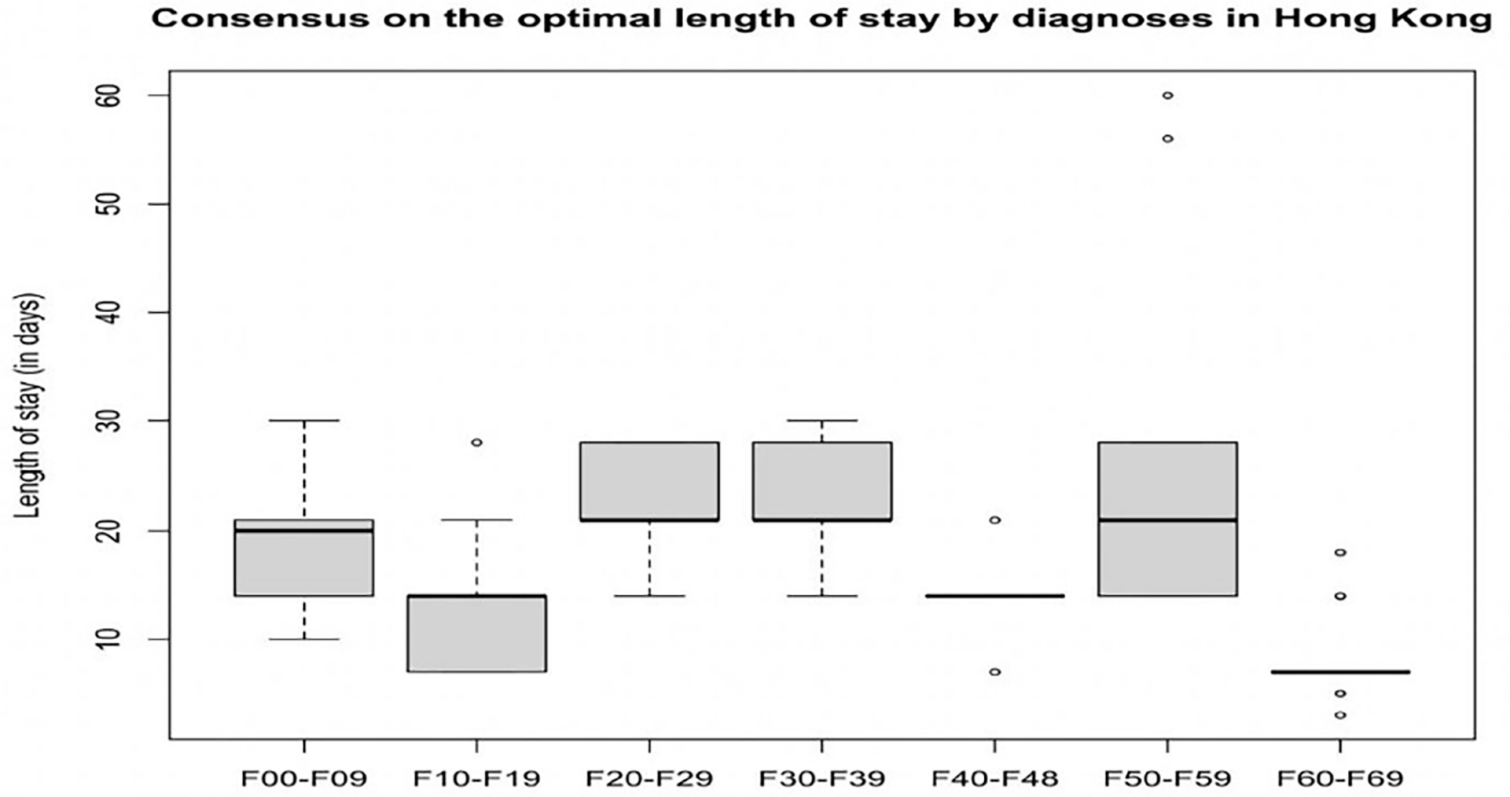
Expert consensus on the optimal length of stay by diagnosis in Hong Kong. F00–F09 = organic mental disorders; F10-F19 = substance use disorders; F20–F29 = schizophrenia-spectrum disorders; F30–F39 = mood disorders; F40–F48 = neuroses; F50–F59 =behavioural syndrome; F60–F69 = personality disorders.

Supplementary Table 2 lists the expert consensus on the LoS (in days) by diagnosis in Hong Kong. The medians and IQRs of the LoSs for each diagnosis are as follows: schizophrenia-spectrum disorders (median = 21, IQR = 5.3), mood disorders (median = 21, IQR = 5.3), neuroses (median = 14, IQR = 0), substance use disorders (median = 14, IQR = 6.3), organic mental disorders (median = 20, IQR = 7), behavioural syndromes (median = 21, IQR = 14), and personality disorders (median = 7, IQR = 0). Figure 2 presents these findings in a boxplot for ease of viewing.

### Expert consensus on the nurse-to-patient ratio in Hong Kong

Table 2 summarises the expert consensus on the daytime nurse-to-patient ratio in acute adult psychiatric wards in Hong Kong. The median ratio was 2.5 nurses to 10 inpatients (IQR = 0.9), indicating that the expert panel regarded one nurse looking after four inpatients be an optimal nurse-to-patient ratio for psychiatric inpatient care.

**Table 2:** Expert consensus on the optimal number of psychiatric nurses per 10 service users (in the daytime) in the two rounds of the Delphi survey.

	First round (*n* = 29)	Second round (*n* = 22)
Median (IQR)	2.5 (1.3)	2.5 (0.9)
Minimum	1.3	2
Q1	2	2.1
Q3	3.3	3
Maximum	5	4
Mean (SD)	2.7 (1.2)	2.6 (0.5)

### Expert consensus on factors contributing to the optimal numbers of inpatient psychiatric beds in Hong Kong

In the first round, a total of 375 contextual factors with 350 rationales were provided. After the removal of duplicates, 49 factors remained. In the second round, 11 factors passed the consensus criterion (≥ 85%, i.e., 19 members rated the factors as ‘important’ or ‘essential’). The 11 factors were grouped into two themes in Table 3. The two themes identified were “Mental health service needs” and “Mental health service provision and support.” To correct for the low base effect, we changed the consensus criterion from 85 to 75% (i.e., factors were considered consensus if 17 members rated them as ‘important’ or ‘essential’) for the sensitivity analysis. An additional theme, “Community mental health service development,” and three additional factors are included in Table 3, as indicated in italic text. Most expert panel members agreed that the optimal psychiatric inpatient bed number was determined predominately by service needs, provision, and support rather than solely by community psychiatric service development.

**Table 3:** Expert consensus on factors that are important for determining the optimal number of inpatient psychiatric beds in Hong Kong.

		No. of responses (%), *n* = 22			
Themes	Factors	Important	Essential	Important + Essential	Met consensus criterion?
**Mental health service needs**	Needs of mental health services	11 (50)	11 (50)	**22 (100)**	***Y***
	Number of new cases (new to mental health service) in the Specialist Outpatient Department and admitted to mental hospitals	15 (68)	6 (27)	21 (96)	***Y***
	Prevalence of severe mental illness	7 (32)	14 (64)	21 (96)	***Y***
	Prevalence of youth mental health problems	16 (73)	5 (23)	21 (96)	***Y***
	Prevalence of substance use	14 (64)	5 (23)	19 (86)	***Y***
	Incidence of suicide	12 (55)	7 (32)	19 (86)	***Y***
	Local population	17 (77)	4 (18)	21 (96)	***Y***
	*Trend in psychiatric inpatient hospitalisation*	12 (55)	6 (27)	18 (82)	*N*
	***Mean percentages***	***59***	***33***	***92***	
**Mental health service provision and support**	Availability of competent healthcare professionals	14 (64)	7 (32)	21 (96)	***Y***
	Availability of effective treatments to control Symptoms	13 (59)	7 (32)	20 (91)	***Y***
	Mental healthcare expenditure	10 (46)	11 (50)	21 (96)	***Y***
	Policy support on mental health	16 (73)	3 (14)	19 (86)	***Y***
	*Availability of subacute psychiatric care, such as partial hospitalisation*	16 (73)	2 (9)	18 (82)	*N*
	*Availability of comorbidity care beds for comprehensive care*	16 (73)	1 (5)	17 (77)	*N*
	***Mean percentages***	***65***	***24***	***88***	
*Community mental health service development*	*Psychiatric community services/primary care*	12 (55)	6 (27)	18 (82)	*N*
	*Support for caregivers*	14 (64)	3 (14)	17 (77)	*N*
	*Public health service planning*	13 (59)	5 (23)	18 (82)	*N*
	***Mean percentages***	***59***	***21***	***80***	

Bold text in themes, meeting a priori consensus; italic text in themes/factors, meeting a posteriori consensus; bold value in no. of responses.

### Expert consensus on recommendations for achieving optimal acute adult inpatient psychiatric services in Hong Kong

In the first round, there were a total of 424 contextual factors with 392 rationales provided. After the removal of duplicates, 74 factors remained. In the second round, 16 factors passed the consensus criterion (≥ 85%, i.e., 19 members agreed or strongly agreed that the factors were important). The 16 factors are grouped under three themes in Table 4. The three themes identified were “Identifying the risks for psychiatric admission,” “Quality psychiatric inpatient service provision,” and “Service evaluation.” To correct for the low base effect, we changed the consensus criterion from 85 to 75% (i.e., factors were accepted if 17 members agreed or strongly agreed that they were important) for the sensitivity analysis. No new theme was created, but five additional factors were added and are presented in Table 4 in italic. Four factors were agreed upon by all expert panel members, of which three were staff-related concerns, and one is a patient-related concern. However, five out of six factors were patient-related concerns in the theme of “Service evaluation.”

**Table 4:** Expert consensus on factors that are important for achieving optimal acute adult inpatient psychiatric care.

		No. of responses (%), *n* = 22			
Themes	Factors	Agree	Strongly agree	Agree + Strongly agree	Met consensus criterion?
Identifying the risks for psychiatric admission	Suboptimal symptom control	13 (59)	7 (32)	20 (91)	***Y***
	Poor medication compliance	8 (36)	13 (59)	21 (96)	***Y***
	Lack of family support or dysfunctional family functioning	16 (73)	6 (27)	**22 (100)**	***Y***
	Stressful life events	15 (68)	5 (23)	20 (91)	***Y***
	*Poor insight*	8 (36)	10 (45)	18 (82)	*N*
	***Mean percentages***	***54***	***37***	***92***	
Quality psychiatric inpatient service provision	Safe and spacious ward environment with sufficient facilities	10 (46)	11 (50)	21 (96)	***Y***
	Caring work culture	8 (36)	14 (64)	**22 (100)**	***Y***
	Structured ward activity (leisure or occupational activities)	13 (59)	7 (32)	20 (91)	***Y***
	Clear and timely treatment plan	14 (64)	7 (32)	21 (96)	***Y***
	Multi-disciplinary approach	11 (50)	8 (36)	19 (86)	***Y***
	Psychotropic medications	9 (41)	12 (55)	21 (96)	***Y***
	Quality clinical supervision	14 (64)	7 (32)	21 (96)	***Y***
	Sufficient manpower	8 (36)	14 (64)	**22 (100)**	***Y***
	Competence level of staff	13 (59)	9 (41)	**22 (100)**	***Y***
	Supportive leadership	14 (64)	7 (32)	21 (96)	***Y***
	Family involvement	13 (59)	6 (27)	19 (86)	***Y***
	***Mean percentages***	***53***	***42***	***95***	
Service evaluation	Staff-to-service user ratio	11 (50)	8 (36)	19 (86)	***Y***
	*Re-admission rate*	12 (55)	6 (27)	18 (82)	*N*
	*Interval of re-admission*	12 (55)	6 (27)	18 (82)	*N*
	*Patient satisfaction*	11 (50)	6 (27)	17 (77)	*N*
	*Service users’ understanding of their illness, symptoms, and treatment*	12 (55)	5 (23)	17 (77)	*N*
	*Service users’ quality of life*	14 (64)	3 (14)	17 (77)	*N*
	***Mean percentages***	***55***	***26***	***80***	

Bold text in themes, meeting a priori consensus; italic text in themes/factors, meeting a posteriori consensus; bold value in no. of responses.

## Discussion

This Delphi survey represents the first attempt to determine the optimal acute adult psychiatric service and the optimal number of adult psychiatric beds in an Asian region. Multidisciplinary expert consensuses on the optimal number of psychiatric beds, optimal nurse-to-patient ratio, optimal LoS by diagnosis, and recommendations for inpatient bed numbers and the optimal acute adult psychiatric service in Hong Kong were established. The optimal median number of psychiatric beds was 68 per 100,000 population. This consensus proposes 39% more beds than currently available in Hong Kong. However, the current bed numbers were in line with the consensus on the minimum required bed numbers of 50 per 100,000 population. The median of the optimal nurse-to-patient ratio was 1:4, which requires more resources than the minimum ratio of 1:6 advocated by SPSC & ICN (15) and legislated by California (16, 17), respectively. The median of the optimal LoS (in days) for schizophrenia-spectrum disorders, mood disorders, neuroses, substance use disorders, organic mental disorders, behavioural syndromes, and personality disorders were 21, 21, 14, 14, 20, 21, and 7, respectively, which were different from that of OECD average LoS data retrieved from 26 countries in 2022, particularly schizophrenia (median = 36 days) and mood disorders (median = 18.9 days) (48). Two and three themes are identified as factors that contribute to optimal inpatient bed numbers and recommendations for the optimal acute adult psychiatric service in Hong Kong.

In the current study, the medians of the optimal and minimum numbers of beds were 68 and 50 per 100,000 population, higher than 60 and 40 per 100,000 population reported as findings from a worldwide Delphi study (5). This may be partly attributable to sociodemographic and cultural differences and crowded living environments with only 172 square feet per capita floor area of accommodation of domestic households in Hong Kong (49). Hong Kong has a population density of 6,740 people per square kilometer (50) and is ranked the fourth most densely populated city in the world (51). The crowded living environment may make caring for people with serious mental illnesses at home and community reintegration more difficult, leading to increased family conflicts as well as a need for unplanned and frequent psychiatric readmissions. High bed-population ratios are also seen in Japan (258 beds per 100,000 population) and South Korea (128 beds per 100,000 population), the other two high-income East Asian countries (10). The frequent use of psychiatric beds might reflect help-seeking patterns in East Asian high-income societies. Others argue that the stagnation of the deinstitutionalisation process in Asia, generally starting from the 1990s to 2000s, was partly due to decolonisation, public stigma, family readiness and acceptance, and mental health policy and leadership (52–55). However, a recent regional study involving ten Southeast Asian countries, most being from lower middle-income countries, revealed that the median number of psychiatric hospital beds was 2.93 per 100,000 populations and was increased by 94% in the recent three decades (56). Hudson (57) revealed that the estimated number of needed psychiatric hospital beds should be 62 per 100,000 population (95% CI: 52–72 per 100,000 population) for high-income countries, adjusting for rates of psychiatric disability, national community mental health services and socioeconomic factors and that about 70% of the 166 nations did not meet the needed bed number (i.e., actual bed numbers not falling in the width of the estimated 95% CI) calculated in the predictive model. A recent worldwide meta-analysis of psychiatric bed needs estimates revealed considerably higher needs estimated than the available beds in most countries from 2000 onwards (58).

McBain et al. (59) and O’Reilly et al. (6) proposed that psychiatric bed estimation should be driven by data in addition to expert consensus. Such data should include real-world clinical data on psychiatric service needs (e.g., occupancy rates, LoS, readmission rates and waitlist volumes) and trends and changes in epidemiological data and demographic composition, as this would ensure that psychiatric inpatient service demand and supply are aligned. These factors were partly addressed by the expert panel members in their consideration of the contextual factors determining the optimal number of psychiatric beds in Hong Kong. The themes generated were the mental health service needs and provision, considering community psychiatric developments in relation to bed numbers. However, balancing inpatient and community psychiatric services whilst considering various factors would need to be a dynamic process requiring good quality national data collections.

This expert consensus in Hong Kong identified 16 key factors for achieving optimal acute adult psychiatric inpatient care, including a spacious ward environment, a caring work culture, sufficient manpower, and a sufficient competence level among staff. All panel members identified family dysfunction as a risk factor for psychiatric admission and 86% of the panel agreed that family involvement was a quality indicator. The expert opinions were also supported by evidence of the importance of family interventions for individuals with mental health challenges and their family caregivers (60–64). With 64% agreement, structured psychotherapeutic intervention programmes did not meet the consensus criteria for a quality factor. In Hong Kong, it may be partly explained by the lack of psychotherapeutic skills among frontline healthcare professionals and the high workload in the psychiatric inpatient setting. Psychological interventions in an adult inpatient setting have been advocated by multidisciplinary professionals in the UK (65, 66). The need to improve communication skills and therapeutic interactions with service users and family caregivers by psychiatric nurses has been emphasised in a qualitative study in the UK (67). Apart from psychological support to improve acute psychiatric services, it was proposed that increased collaboration with community NGOs or stakeholders, the promotion of service user-led and co-produced programmes and making good use of telepsychiatry were future trends in the development of acute psychiatric services (30). However, the staff-to-patient ratio was the only consensus on the theme of service evaluation in this current study. Numerous research reports supported that the staff-to-patient ratio was positively associated with patient outcomes in Europe (68), Australia (69), Chile (70), and South Korea (71).

A strength of the study is that it strongly represents the views of psychiatric nurses in the public inpatient service system in Hong Kong. The sample size of the study is also comparable with similar previous studies (5, 9). However, the response rate was only marginally satisfactory, which might be related to the heavy workload experienced during the COVID-19 pandemic period (72), and the overall moderate attrition rate of the two-round surveys may incur selection bias, retaining participants who were more willing to be engaged in the future academic and clinical psychiatric development. A limitation of the study is that eligible participants were conveniently identified by three healthcare professionals (i.e., a psychiatrist and two psychiatric nurses) in their workplaces, which might limit access and participation by other healthcare professionals, such as clinical psychologists, occupational therapists and physiotherapists. The estimation of the psychiatric bed shortage was based on individual participants’ interpretation, which might incur interpretation bias. In addition, the views of service users, particularly peer support workers, and family caregivers were not included. Finally, the results of the study may be more valid in the local setting with limited generalizability.

## Conclusion

This is the first expert consensus study using the Delphi method on the optimal number of psychiatric beds and quality factors of acute adult psychiatric inpatient services in Hong Kong. Results suggested that the optimal number of inpatient psychiatric beds is 68 per 100,000 population. Sixteen key factors were identified for optimal psychiatric inpatient care, including caring work culture and sufficient manpower. This study can inform further strategies for monitoring and service development of acute adult psychiatric inpatient care in Hong Kong. The current study can be considered a starting point for more extensive studies considering the viewpoints of other stakeholders including people with lived experience and service users.

## Data Availability

The raw data supporting the conclusions of this article will be made available by the authors, without undue reservation.
