# Supplementary material for: A multidisciplinary expert consensus on optimal acute adult psychiatric inpatient care in Hong Kong
Source: Front Public Health. 2025 Dec 15;13:1719409. doi: 10.3389/fpubh.2025.1719409 (PMC12745207; doi:10.3389/fpubh.2025.1719409)
Supplement: Supplementary file 1 [file Table_1.doc]

***Supplementary Table 1.*** Expert consensus on the optimal, minimum, and shortage in numbers of psychiatric beds in the two rounds of the Delphi survey

|  | **First round (*n* = 25)*** | | | | | **Second round (*n* = 22)** | | | | |
| --- | --- | --- | --- | --- | --- | --- | --- | --- | --- | --- |
|  | **Optimal** | **Minimum** | **Mild**  **shortage** | **Moderate**  **shortage** | **Severe**  **shortage** | **Optimal** | **Minimum** | **Mild**  **shortage** | **Moderate**  **shortage** | **Severe**  **shortage** |
| **Median (IQR)** | 65  (53) | 49  (30) | 41  (25) | 34  (29) | 27  (27) | 68  (10) | 50  (7) | 41  (7) | 34  (10) | 27  (10) |
| **Minimum** | 27 | 10 | 12 | 10 | 3 | 34 | 30 | 30 | 20 | 14 |
| **Q1** | 55 | 30 | 30 | 20 | 14 | 65 | 48 | 40 | 31 | 21 |
| **Q3** | 108 | 60 | 55 | 49 | 41 | 75 | 55 | 47 | 41 | 32 |
| **Maximum** | 700 | 545 | 681 | 500 | 700 | 100 | 60 | 55 | 49 | 44 |
| **Mean (SD)** | 148  (204) | 105  (158) | 97  (154) | 70  (100) | 47  (61) | 71  (13) | 50  (8) | 43  (6) | 36  (8) | 27  (8) |

*Four responses were not obtained in the first round of the Delphi survey.

Footnote: IQR = interquartile range; SD = standard deviation

***Supplementary Table 2.*** *Expert consensus on the optimal length of stay (days) by diagnosis in Hong Kong* in the two rounds of the Delphi survey

|  | **First round (*n* = 29)** | | | | | | | **Second round (*n* = 22)** | | | | | | |
| --- | --- | --- | --- | --- | --- | --- | --- | --- | --- | --- | --- | --- | --- | --- |
|  | **F00–F09** | **F10–F19** | **F20–F29** | **F30–F39** | **F40–F48** | **F50–F59** | **F60–F69** | **F00–F09** | **F10–F19** | **F20–F29** | **F30–F39** | **F40–F48** | **F50–F59** | **F60–F69** |
| **Median (IQR)** | 14  (16) | 14  (7) | 21  (14) | 21  (14) | 14  (10) | 20  (14) | 7  (10) | 20  (7) | 14  (6) | 21  (5) | 21  (5) | 14  (0) | 21  (14) | 7  (0) |
| **Minimum** | 7 | 3 | 7 | 7 | 3 | 3 | 3 | 14 | 7 | 14 | 14 | 7 | 14 | 3 |
| **Q1** | 14 | 7 | 14 | 14 | 10 | 14 | 5 | 14 | 8 | 21 | 21 | 14 | 14 | 7 |
| **Q3** | 30 | 14 | 28 | 28 | 20 | 28 | 15 | 21 | 14 | 26 | 26 | 14 | 28 | 7 |
| **Maximum** | 60 | 30 | 60 | 60 | 30 | 150 | 60 | 30 | 28 | 28 | 30 | 21 | 60 | 18 |
| **Mean (SD)** | 22  (17) | 13  (8) | 24  (13) | 23  (12) | 14  (7) | 28  (29) | 13  (12) | 19  (6) | 13  (5) | 23  (4) | 23  (4) | 14  (2) | 24  (12) | 8  (3) |

Footnote: F00–F09 = organic mental disorders; F10–F19 = substance abuse disorders; F20–F29 = schizophrenia-spectrum disorders; F30–F39 = mood disorders; F40–F48 = neuroses; F50–F59 = behavioural syndromes; F60–F69 = personality disorders; IQR = interquartile range; SD = standard deviation
